# *Toll-like receptor 9* polymorphisms are associated with severity variables in a cohort of meningococcal meningitis survivors

**DOI:** 10.1186/1471-2334-12-112

**Published:** 2012-05-11

**Authors:** Marieke S Sanders, Gijs TJ van Well, Sander Ouburg, Servaas A Morré, A Marceline van Furth

**Affiliations:** 1 Laboratory for Immunogenetics, Department of Medical Microbiology and Infection Control, VU University Medical Center, Amsterdam, 1007 MB, The Netherlands; 2 Department of Pediatric Infectious Diseases, Immunology and Rheumatology, VU University Medical Center, Amsterdam, 1007 MB, The Netherlands; 3 Department in Surgery, Antonius Hospital Nieuwegein, Nieuwegein, The Netherlands; 4 Department of Pediatrics, Maastricht University Medical Center (MUMC+), Maastricht, 6202 AZ, The Netherlands

## Abstract

****Background**:**

Genetic variation in immune response genes is associated with susceptibility and severity of infectious diseases. Toll-like receptor (TLR) 9 polymorphisms are associated with susceptibility to develop meningococcal meningitis (MM). The aim of this study is to compare genotype distributions of two *TLR9* polymorphisms between clinical severity variables in MM survivors.

****Methods**:**

We used DNA samples of a cohort of 390 children who survived MM. Next, we determined the genotype frequencies of *TLR9* -1237 and *TLR9* +2848 polymorphisms and compared these between thirteen clinical variables associated with prognostic factors predicting adverse outcome of bacterial meningitis in children.

****Results**:**

The *TLR9 -*1237 TC and CC genotypes were associated with a decreased incidence of a positive blood culture for *Neisseria (N.) meningitidis* (*p* = 0.014, odds ratio (OR) 0.5. 95% confidence interval (CI) 0.3 – 0.9). The *TLR9 +*2848 AA mutant was associated with a decreased incidence of a positive blood culture for *N. meningitidis* (*p* = 0.017, OR 0.6, 95% CI 0.3 – 0.9). Cerebrospinal fluid (CSF) leukocytes per μL were higher in patients carrying the *TLR9 -*1237 TC or CC genotypes compared to carriers of the TT wild type (WT) (*p* = 0.024, medians: 2117, interquartile range (IQR) 4987 versus 955, IQR 3938). CSF blood/glucose ratios were lower in *TLR9 -*1237 TC or CC carriers than in carriers of the TT WT (*p* = 0.017, medians: 0.20, IQR 0.4 versus 0.35, IQR 0.5). CSF leukocytes/μL were higher in patients carrying the *TLR9 +*2848 AA mutant compared to carriers of GG or GA (*p* = 0.0067, medians: 1907, IQR 5221 versus 891, IQR 3952).

****Conclusions**:**

We identified TLR9 genotypes associated with protection against meningococcemia and enhanced local inflammatory responses inside the central nervous system, important steps in MM pathogenesis and defense.

## Background

The susceptibility, severity and prognosis of infectious diseases depend on the ability of the host immune system to respond to pathogens. Genetic variation of immune response genes is associated with susceptibility to and severity of infectious diseases [1]. Bacterial meningitis (BM) is a serious and life-threatening infectious disease of the central nervous system (CNS). Despite adequate antibiotic treatment and immunization strategies, mortality remains high, especially in developing countries [2,3]. *Neisseria (N.) meningitidis* is a common causing pathogen of BM, both in the Western world as in developing countries. The clinical course of meningococcal meningitis (MM) is highly diverse and depends both on pathogen characteristics as on the individual immune response of the affected patient. Host-bacteria interactions are crucial in defense against MM [4]. Acquisition of *N. meningitidis* may lead to bacterial colonization in one patient and to fatal MM or meningococcal septic shock in the other. Survivors of BM have a high risk to develop neurological sequelae, ranging from subtle learning and behavioral disorders to deafness, paresis, and severe encephalopathy [5-7]. Innate immunity is of particular importance as first line of defense since it quickly senses pathogen invasion by pattern recognition and subsequently initiates the immune response. Toll-like receptors (TLRs) are a class of pathogen recognition receptors (PRRs) that are key players of innate immunity. It becomes increasingly clear that TLR mediated meningeal inflammation is a pivotal factor for meningitis associated tissue damage [8]. TLR9 is an intracellular PRR which recognizes unmethylated Cytosine-phosphate-Guanine (CpG) motives in pathogen DNA [9]. Meningococcal CpG DNA enters TLR9 expressing cells by endocytosis and then binds to TLR9. A cascade of intracellular receptor signaling via myeloid differentiation protein 88 (MyD88) induces activation of transcription of nuclear factor kappa B (NFkB) resulting in the production of cytokines and chemokines [10]. TLR9 is present in phagocytosing microglia and antigen presenting astrocytes inside the CNS, cells responsible for adequate immune responses in this compartment [11]. In a previous study we demonstrated that the *TLR9* +2848 SNP is associated with a decreased susceptibility to MM [12]. Recent studies showed that carriage of the *TLR9 -*1237 C variant allele creates a potential nuclear factor kappa B (NFκB) binding site that increases the transcriptional activity of *TLR9* and enhances cellular production of pro-inflammatory cytokines [13].

The purpose of this study is to compare the genotype distributions of *TLR9* -1237 and *TLR9* +2848 single nucleotide polymorphisms (SNPs) between thirteen clinical severity variables in order to identify patients at risk for severe disease and sequelae.

## **Methods**

The study population consists of 390 Dutch Caucasian children who survived MM. These patients were identified by the Dutch Reference Laboratory for Bacterial Meningitis. The diagnosis of MM was based on a positive cerebrospinal fluid (CSF) culture with *N. meningitidis* or meningococcal antigens in the CSF. A total of 560 children were asked to participate in the study and to return a sterile swab after collecting their buccal DNA, of whom 390 patients (70%) returned a buccal swab and informed consent form. Patients were diagnosed between January 1990 and December 1995 and this cohort was previously described in detail by Koomen *et al.*[6,7]. A similar validation cohort of 76 children developed BM between 1997 and 2001 [14]. Data for our study were collected in the period from 2006 till 2010. Median age at infection was 2,5 (range 0.1 – 9.5) years, 46.5% were female, 53.5% were male. Data on medical history, physical examination, clinical course during hospitalization, and laboratory results were gathered from the medical records of all patients.

Children with ‘complex onset’ of meningitis (defined as meningitis secondary to immune deficiency states, cranial trauma, CNS surgery, and CSF shunt infections), relapsing meningitis, or meningitis in the neonatal period were excluded.

The Medical Ethical Committee of the VU University Medical Center, Amsterdam, The Netherlands approved this study.

*TLR9* -1237 (rs5743836) and *TLR9* +2848 (rs352140) SNPs were analyzed in buccal DNA by TaqMan analysis using the standard TaqMan protocol. The AbiPrism® 7000 Sequence Detection System (Applied Biosystems, UK) was used to obtain data. Primers and probes we used have been described previously [12]. The two *TLR9* SNPs were chosen based on a study by Lazarus *et al.* In three ethnic groups they found 20 *TLR9* SNPs. A set of four frequent *TLR9* SNPs (*TLR9 -*1486, *TLR9 -*1237, *TLR9 +*1174 and *TLR9 +*2848) accounted for more than 75% of all chromosomes in all three populations. Genotyping of both *TLR9 -*1237 T > C and *TLR9 +*2848 G > A allows all four locus haplotypes to be distinguished [15].

We performed a literature search to identify severity variables. We used clinical variables: duration of clinical illness before admission, rectal temperature, convulsions, level of consciousness at admission, ICU admission, main clinical diagnosis at discharge (meningitis or meningitis with sepsis), and post meningitis hearing loss. Convulsions were defined as convulsions reported before or at admission or during hospitalization. Post meningitis hearing loss was defined as > 25 dB perceptive hearing loss that was not present before meningitis occurred. Laboratory variables at admission included: blood culture results, CSF leukocyte numbers, CSF/blood glucose ratios, CSF protein concentrations, blood leukocyte numbers, and C-reactive protein (CRP) concentrations in serum. The selected variables were in accordance with a recent systematic review summarizing the evidence regarding prognostic factors predicting death or sequelae after BM in children [16]. We distinguish continuous and dichotomous variables. Continuous variables were dichotomized according to clinical relevant cut off points known from the literature i.e. duration of clinical illness before admission > 2 days, rectal temperature ≥ 38°C, CSF leukocyte numbers > 600 and > 1000 [17,18], CSF blood/glucose ratio ≤ 0.4, CSF protein concentration > 0.7 g/L, blood leukocytes > 20x10^9 g/L, and serum CRP concentration > 100 mg/L [16].

Within selected severity groups, we compared the distribution of *TLR9 -*1237 and +2848 genotypes. For statistical analysis, SPSS for Windows 17.0 and Graphpad Prism 5 were used. Genotype distributions were checked for deviations of the Hardy-Weinberg equilibrium (HWE). Recessive and dominant models were used to model the relations between genotype distributions and clinical variables. Histograms were used to assess normality of the clinical variables. T-tests, Mann–Whitney U tests, and χ^2^ test or Fisher’s exact tests were used where appropriate. Outliers (< 4%) were excluded by the Grubbs’ test (*p* < 0.01) before continuous testing. *P* values < 0.05 were considered statistically significant.

## **Results**

Table 1 shows the distribution and characteristics of the severity variables in the study population. Differences in numbers of patients are due to missing or non-determined data in patient records.

**Table 1:** Distribution and characteristics of 13 severity variables in children with meningococcal meningitis

Severity variable					Total
Continuous variable	Median		Range		N
Duration of clinical illness before admission (days)	1.7		0.5 – 13.0		324
Rectal temperature (°C)	39.1		35.0 – 41.8		358
CSF leukocytes (/μL)	1 227		0.0 – 12 081		354
CSF/blood glucose ratio	0.34		0 – 1.77		280
CSF protein concentrations (g/L)	1.4		0.01 – 9.33		317
Blood leukocytes (x10^9 g/L)	16.2		0.8 – 57.2		382
C-reactive protein (CRP) (mg/L)	129		0 – 768		229
Dichotomous variable	N	N (%)		N (%)	N
*N. meningitidis* in blood culture	No	187 (54)	Yes	161 (46)	348
Convulsions	No	355 (91)	Yes	35 (9)	390
Level of consciousness at admission	Normal	133 (35)	Disturbed	242 (65)	375
ICU-admission	No	302 (78)	Yes	87 (22)	389
Main clinical diagnosis at discharge: (MM without/with sepsis)	No sepsis	219 (56)	Sepsis	171 (44)	390
Post meningitis hearing loss	No	375 (96)	Yes	15 (4)	390
Academic and behavioural limitations	No	90 (61)	Yes	57 (39)	147

*Abbreviations: CSF*: cerebrospinal fluid, *ICU*: intensive care unit, *MM*: meningococcal meningitis. Different numbers within groups are due to missing or non determined data in patient records.

**Figure 1:**
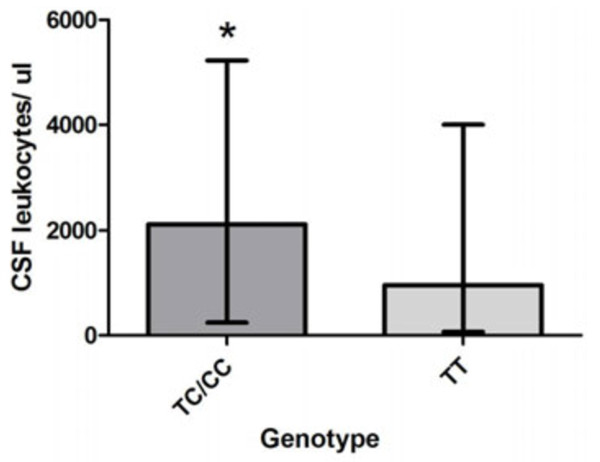
**(A) Comparison of CSF leukocytes per μL in*TLR9 -*1237 TC/CC carriers versus wild type (WT) carriers.** Carriers of *TLR9 -*1237 TC/CC had significantly higher CSF leukocyte numbers compared to carriers of the TT WT (medians: 2117, interquartile range (IQR) 4987 versus 955, IQR 3938). Mann–Whitney *U* test, * p = 0.024. *Abbreviatons:* TLR: Toll-like receptor, SNP: single nucleotide polymorphism, CSF: cerebrospinal fluid, ul: microliter. (**B**) Comparison of CSF/blood glucose ratios in *TLR9 -*1237 TC/CC carriers versus WT carriers. Carriers of *TLR9 -*1237 TC/CC genotypes display significant lower ratios compared to carriers of the TT WT (medians: 0.20, IQR 0.4 versus 0.35, IQR 0.5). Mann–Whitney *U* test, * *p* = 0.017. *Abbreviatons:* TLR: Toll-like receptor, SNP: single nucleotide polymorphism, CSF: cerebrospinal fluid, ul: microliter. (**C**) Comparison of CSF leukocytes per μL in *TLR9 +*2848 AA mutant carriers versus GG/GA carriers. Carriers of the *TLR9* AA mutant display significantly higher CSF leukocyte levels compared to GG/GA carriers (medians: 1907, IQR 5221 versus 891, IQR 3952). Mann–Whitney *U* test, ** *p* = 0.0067. *Abbreviatons: TLR*: Toll-like receptor, *SNP*: single nucleotide polymorphism, *CSF*: cerebrospinal fluid, ul: microliter.

Continuous variables i.e. duration of clinical illness, rectal temperature, CSF leukocyte numbers, CSF/blood glucose ratios, CSF protein concentrations, blood leukocytes, and serum CRP concentrations were compared between carriers of wild type (WT) alleles and mutant alleles in MM patients for *TLR9 -*1237 and *TLR9 +*2848 respectively. Figure 1A shows that CSF leukocyte numbers were significantly higher in MM patients carrying the *TLR9 -*1237 TC or CC genotypes compared to carriers of the TT WT genotype (median 2117, interquartile range (IQR) 4987 versus median 955, IQR 3938).

CSF/blood glucose ratios were significantly lower in *TLR9 -*1237 TC or CC carriers than in carriers of the TT WT (*p* = 0.017, median 0.20, IQR 0.4 versus median 0.35, IQR 0.5) (Figure 1B). CSF leukocytes/μL were significantly higher in patients carrying the *TLR9 +*2848 AA mutant compared to children with genotype GG or GA. (*p* = 0.0067, median 1907, IQR 5221 versus median 891, IQR 3952) (Figure 1C). There was no significant difference in CSF/blood glucose ratios for *TLR9 +*2848 genotypes (results not shown).

No significant differences in genotype distributions of −1237 and +2848 SNPs were found for the other continuous variables (data not shown).

Dichotomous variables, i.e. blood culture, convulsions in patient history, level of consciousness at admission, ICU admission, sepsis and hearing loss and the dichotomized continuous variables (as described above) were compared between WT carriers and mutant carriers. *TLR9 -*1237 TC or CC mutant carriers were compared to the TT WT carriers. Significant associations are shown in Table 2. The *TLR9 -*1237 TC and CC genotypes were associated with a decreased incidence of a positive blood culture for *N. meningitidis* (*p* = 0.014, odds ratio (OR) 0.5, 95% confidence interval (CI) 0.3 – 0.9). These genotypes were also associated with CSF leukocyte levels > 1000 per μL (*p* = 0.029, OR 1.7, 95% CI 1.1 – 2.8), and with a CSF/blood glucose ratio ≤ 0.4 (*p* = 0.015, OR 2.0; 95% CI 1.1 – 3.6).

**Table 2:** ***TLR9 -*1237 SNPs and severity variables in meningococcal meningitis patients (*p*** **< 0.05)**

Severity variable	*TLR9 -*1237 N (%)				P	OR	95% CI
	TT	TC	CC	Total			
*N. meningitidis* in blood culture							
Positive	127 (80)	29 (18)*	3 (2)*	159	0.014	0.5	0.3 – 0.9
Not detected	124 (68)	52 (29)*	6 (3)*	182			
Total	251	81	9	341			
CSF leukocytes per μL							
≤ 1000	129 (78)	34 (21)*	2 (1)*	165	0.029	1.7	1.1 – 2.8
> 1000	127 (68)	52 (29)*	6 (3)*	183			
Total	253	87	8	348			
CSF/blood glucose ratio							
≤ 0.4	114 (69)	46 (28)*	6 (4)*	166	0.015	2.0	1.1 – 3.6
> 0.4	90 (82)	18 (16)*	2 (2)*	110			
Total	204	64	8	276			

* = significance *p* < 0.05.

*Abbreviations: TLR*: Toll-like receptor, *OR*: Odds ratio, 95% *CI*: 95% confidence interval, *CSF*: cerebrospinal fluid. Different numbers within groups are due to missing or non determined data in patient records.

No significant differences in *TLR9 -*1237 genotype distribution were observed between the groups classified by the other severity variables (data not shown).

*TLR9 +*2848 mutant carriers were compared with WT carriers. Significant associations are shown in Table 3. *TLR9 +*2848 AA was associated with a decreased incidence of a positive blood culture (*p* = 0.017, OR 0.6, 95% CI 0.3 – 0.9). The *TLR9 +*2848 AA mutant was also significantly more present in children with > 600 and > 1000 leukocytes per μL (*p* = 0.028, OR 1.7, 95% CI 1.1 – 2.9 and *p* = 0.005, OR 2.0, 95% CI 1.2 – 3.2 respectively).

**Table 3:** ***TLR9 +*2848 SNPs and severity variables in meningococcal meningitis patients (*p*** **< 0.05)**

Severity variable	*TLR9 +*2848 N (%)				P	OR	95% CI
	GG	GA	AA	Total			
*N. meningitidis* in blood culture							
Positive	42 (27)	80 (51)	36 (23)*	158	0.017	0.6	0.3 – 0.9
Negative	40 (22)	79 (43)	63 (35)*	182			
Total	82	159	99	340			
CSF leukocytes/μL							
≤ 600	36 (27)	66 (50)	30 (23)*	132	0.028	1.7	1.1 – 2.9
> 600	50 (24)	91 (43)	72 (34)*	213			
Total	86	157	102	345			
≤ 1000	41 (25)	85 (53)	36 (22)**	162	0.005	2.0	1.2 – 3.2
> 1000	45 (25)	72 (39)	66 (36)**				
Total	86	157	102				

* = significance *p* < 0.05, ** = significance *p* < 0.01

*Abbreviations: TLR*: Toll-like receptor, *OR*: Odds ratio, 95% *CI*: 95% confidence interval, *CSF*: cerebrospinal fluid. Different numbers within groups are due to missing or non determined data in patient records.

No significant differences for *TLR9 +*2848 genotype distribution were observed between the groups classified by the other severity variables (data not shown).

Table 4 shows that *TLR9* haplotype I was very significantly associated with blood cultures positive for *N. meningitidis* (*p* = 0.001, OR 1.5, 95% CI 1.1 – 2.0). Haplotype I was also significantly associated with decreased CSF leukocytes (less than 1000/μL: *p* = 0.03; OR 0.7 95% CI 0.5–1.0). No significant differences in haplotype distribution were observed for other severity variables (data not shown).

**Table 4:** ***TLR9*haplotypes and severity variables in meningococcal meningitis patients (*p*** **< 0.05)**

Severity variable	TLR9 haplotype¹ N (%)					P	OR	95%	CI
	I	II	III	IV	Total				
*TLR9*-1237	T	T	C	C	2 N				
*TLR9* + 2848	G	A	A	G					
*N. meningitidis* in blood culture									
Positive	162 (51)**	119 (38)	33 (10)	2 (1)	316	0.001	1.5	(1.1 2.0)	–
Negative	148 (41)**	147 (41)	59 (16)	4 (1)	358				
Total	310	266	92	6	674				
CSF leukocytes per μL									
≤ 1000	164 (50)*	122 (38)	36 (11)	2 (1)	324	0.03	0.7	(0.5 1.0)	–
>1000	154 (42)*	144 (40)	60 (17)	4 (1)	362				
Total	31	266	96	6	686				

¹ haplotypes as defined by Lazarus et al. [12].

* = significance *p* < 0.05. ** = significance *p* < 0.01.

*Abbreviations: TLR*: Toll-like receptor *CSF*: cerebrospinal fluid, *OR*: odds ratio.

Different numbers within groups are due to missing or non determined data in patient records.

## **Discussion**

We demonstrate that the *TLR9* -1237 and +2848 SNPs are associated with severity variables in a cohort of MM survivors. In order to assess the biological consequence of this statistical association we focus on the essential steps in BM pathogenesis and the recognition of *N. meningitidis* by TLR9. Meningococci colonize the nasopharynx and may penetrate the mucosal barrier of the upper respiratory tract by transcellular passage of epithelial cells [19]. During this process meningococci are recognized by intracellular TLR9 of sinonasal epithelial cells [20]. After passage of this epithelial barrier meningococci are able to pass directly from the nasopharynx to meninges through the olfactory nerve system but more frequently they will enter the bloodstream [21]. Survival of bacteria within the circulation is a prerequisite for meningeal invasion. Complement-mediated opsonophagocytosis of *N. meningitidis* leads to activation of phagocytosing cells via TLR9 [22]. Upon survival in the bloodstream meningococci may attach to and traverse the blood–brain barrier by endocytosis, they will multiply in the subarachnoidal space and are recognized by astrocytes and microglia, dendritic cells and macrophages of the brain respectively and in direct contact with the CSF [23]. After being phagocytosed, meningococcal DNA motifs activate endosomal TLR9 and subsequent signal transduction occurs, stimulating the production of cytokines inside the CNS and chemokines leading to leukocyte recruitment towards the CNS [24,25].

**Figure 2:**
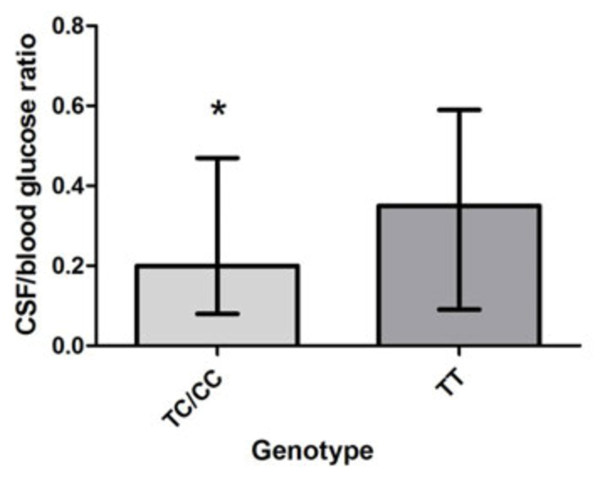
**Schematic diagram of *Toll-like receptor*-9 (*TLR9)*-1237 and +2848 SNP positions and transcription factor (TF) binding sites in the*TLR9 *promotor region. ***In silico* analyses show that the C allele at position −1237 creates extra putative binding sites for Nuclear Factor kappa B (NFκB), avian reticuloendotheliosis viral oncogene homolog A (RelA) and signal transducer and activator of transcription (STAT3). *RelA is able to bind to NFkB to form the NFkB complex. STAT3 is able to activate transcription in the nucleus in response to cytokines.* These processes may upregulate the expression of *TLR9,* altering the TLR9 initiated innate immune response to meningococcal DNA, affecting the clinical severity of meningococcal meningitis (partly adapted from Ng *et al.*, [13]. *Abbreviations: TF*: transcription factor, *R*: A/G, N: any base, Y: C/T, W: A/T, D: A/T/G.

We associated *TLR9* SNPs with protection against meningococcemia, a prerequisite for meningeal invasion, and with elevated CSF leukocyte levels during MM. A decreased incidence of positive blood cultures in children carrying *TLR9* -1237 C allele and *TLR9* +2848 AA genotypes may represent a reduction in the occurrence of secondary bacteremia due to more pronounced host immune response in the CSF. A possible mechanistic explanation and biological consequence is increased NFκB binding to the *TLR9* promotor region, leading to increased transcriptional regulation of *TLR9*[13]. We confirmed this mechanism in our study population using a novel *in silico* regulatory SNP detection method as described by McIntyre *et al.*[26]: The *TLR9* -1237 “C” variant was associated with significantly increased binding of NFκB, avian reticuloendotheliosis viral oncogene homolog A (RelA) and signal transducer and activator of transcription (STAT3) to the *TLR9* -1237 C variant (Figure 2). According to these predictions, the *TLR9* -1237 C allele creates an increased affinity for NFκB which in its turn increases the transcriptional activity of the gene, leading to enhanced production of cytokines and chemokines. This effect was present in stimulated cells, but not under basal conditions, which may explain an association of *TLR9*-1237 with severity, but not susceptibility to MM. Carvalho *et al.* reported that the C allele of TLR9-1237 introduced a new IL-6-dependent transcription factor binding site in the TLR9 promoter. Peripheral blood mononuclear cells (PBMCs) harbouring the TC genotype show higher expression of both TLR9 and IL-6 and increased B-cell proliferation in response to CpG [27]. Another study showed higher serum Interferon gamma levels in children carrying the *TLR9*-1237-C allele with cerebral malaria, indicating that enhanced TLR9 mediated immune responses are also relevant inside the CNS [28]. The +2848 SNP does neither result in an amino acid change nor to the modification of a regulatory site, implying linkage of a functional relevant SNP in the vicinity of this SNP.

In a mouse meningococcal bacteremia model the role of TLR9 in preventing bacteremia was also confirmed. *TLR9* KO mice displayed reduced survival and elevated levels of bacteremia compared to WT mice [22]. We associated *TLR9* polymorphisms with prevention of bacteremia and higher levels of leukocytes in the CSF. The link between systemic inflammation and pleocytosis was previously studied. Intravenous injection of LPS prior to intracisternal LPS injection in rabbits led to impaired pleocytosis, reduced levels of TNF-α, and impaired leukocyte influx into the CNS reflecting an impaired inflammatory response in the CNS [29].

Clinical studies show that the most severely affected patients with MM or septic shock with a rapidly evolving septic shock associated with high mortality have significant lower levels of pleocytosis [30]. Although we found an association of *TLR9* SNPs with bacteremia, no association was found with clinical sepsis. We propose that *TLR9 -1237* and +2848 polymorphisms have a beneficial effect on preventing bacteremia and increase the leukocyte influx in the CNS, reflecting an enhanced immune response inside the CNS.

Although CNS inflammation is necessary to guarantee sterility of the CNS, its injurious properties are also evident. An adequate but balanced inflammatory response inside the CNS is essential in limiting adverse outcome of disease*.* We concluded that *TLR9* polymorphisms have a small but possibly important contribution to warrant balance between beneficiary and injurious effects of inflammation in the CNS. For exact consequences of these SNPs, future studies focussing on *TLR9* SNPs and long term consequences of BM should be performed.

This study does have certain aspects that limit the interpretations. Survivors of MM were retrospectively included in our study. DNA from children with fatal meningitis were not a focus of our study because including survivors of MM allows us to obtain detailed information on follow-up and long term consequences of the disease which are of particular clinical relevance. In addition, the number of patients with fatal meningitis is very low in The Netherlands. The effect of treatment of BM was not incorporated in this study. This effect may not be of great influence since comparable protocols for treatment of BM were used nationwide in the period these children have been admitted to Dutch hospitals, and timing of treatment is divided equally between groups and independent of genotype distribution.

Multidisciplinary efforts are needed in order to bundle and translate genetic studies into beneficial interventions (personalized medicine, risk profiling, disease treatments with better specificity and innovative drug therapies) enforced by the field called Public Health Genomics [31].

We have performed several statistical analyses and therefore also performed corrections for multiple testing. Using the rough false discovery rate (rFDR) would shift the threshold for statistical significance from <0.05 to <0.026, reducing the number of significant associations described in this paper. Using the more conservative Holm-Bonferroni corrections, shifts the threshold to <0.002, resulting in one remaining significant association (*TLR9* haplotype I and *N. meningitidis*). Although the multiple testing corrections reduce the number of statistically significant associations of the exploratory study, the trends that remain give indications for potential roles of the studied variables in the severity of bacterial meninigitis.

More association and functional studies on *TLR9* SNPs, functional consequences and long term effects in the CNS are needed to reveal the exact mechanism causing the differences in clinical course of MM and to obtain genetic traits which can be used for patient profiling and management of meningitis patients.

## **Conclusions**

In this study, *TLR9* -1237 and *TLR9* +2824 mutant genotypes were associated with protection against meningococcemia, an essential prerequisite for meningeal invasion. We also associated these SNPs with enhanced local inflammatory responses inside the CNS. Carriers of the *TLR9* + 2848 mutant genotype have a lower chance of developing meningitis when colonized with *N. meningitidis*. When they eventually do so, they will have more efficient bacterial killing inside the CNS but may also result in a higher chance of developing neurological sequelae.

## **Competing interests**

All authors declare to have no competing interests of any kind regarding this study.

## **Authors’ contributions**

GTJvW and MSS collected the patient data and samples, performed the genotype and statistical analysis and wrote the manuscript. SO supervised the genotype and statistical analysis, performed the *in silico* analysis, and critically revised the manuscript. AMvF and SAM designed and supervised the study and critically revised the manuscript. All authors have read and approved the final version of the manuscript.

## Pre-publication history

The pre-publication history for this paper can be accessed here:

http://www.biomedcentral.com/1471-2334/12/112/prepub
